# W_18_O_49_@EP nanoparticles improve the anti-tumor effect of radiotherapy and photodynamic therapy by avoiding the limitation of hypoxia

**DOI:** 10.3389/fbioe.2022.1060467

**Published:** 2022-11-10

**Authors:** Jianquan Wang, Lingyun Hao, Xinhua Li, Sen Liu

**Affiliations:** ^1^ School of Materials Engineering, Jinling Institute of Technology, Nanjing, China; ^2^ Nanjing Key Laboratory of Optometric Materials and Technology, Nanjing, China; ^3^ Jiangsu Provincial Engineering Research Center for Biomedical Materials and Advanced Medical Devices, Faculty of Mechanical and Material Engineering, Huaiyin Institute of Technology, Huaian, China

**Keywords:** W_18_O_49_, nanoparticles, hypoxia, ROS, RT, PDT, tumor microenvironment

## Abstract

Insufficient oxygen supply at the tumor site and hypoxia caused during tumor treatment lead to a poor therapeutic effect and poor prognosis. Therefore, effectively overcoming the problem of hypoxia in tumors and avoiding hypoxia that compromises the efficacy of the treatment could improve the anti-tumor therapeutic effect. Thus, this study reports the ability of W_18_O_49_@EP nanoparticles to release reactive oxygen species (ROS) during the combined tumor radiotherapy (RT) and photodynamic therapy (PDT). The release of ROS by the nanoparticles during near infrared light (NIR) irradiation was demonstrated by *in vitro* and *in vivo* experiments, realizing an effective PDT without inducing hypoxia. Indeed, the ROS did not derive from the oxygen in the tumor microenvironment but they were released by the nanoparticles. Thus, ROS could improve the therapeutic effect of RT avoiding the problem of hypoxia after RT. Hence, W_18_O_49_@EP nanoparticles greatly improved the anti-tumor effect due to their effectiveness despite the insufficient oxygen supply and hypoxia caused by traditional RT and PDT.

## 1 Introduction

Radiotherapy (RT) is an effective approach in the treatment of tumors, and more than 50% of all cancer patients receive RT (Allen et al., 2017). RT potently kills cells mainly through the damage of DNA by ionization (Atun et al., 2015; De Ruysscher et al., 2019). RT also generates reactive oxygen species (ROS), thereby indirectly leading to DNA damage (De Ruysscher et al., 2019). Another significant effect of ROS is that they trigger apoptosis by inducing stress responses in the subcellular organelles, such as mitochondria and the endoplasmic reticulum, which effectively enhance the cell-killing effect of RT (Rey et al., 2017). However, tumor cells have a strong self-regulation ability of detoxify and eliminate the effect of ROS through the transcription of antioxidant enzymes when the level of ROS is relatively low (Kim et al., 2019). The apoptotic process is activated only when the ROS levels are high enough to inhibit the self-regulation of the cells, thus killing the abnormal cells. Unfortunately, the excessive production of ROS leads to hypoxia, which is the main factor leading of the resistance of tumor cells to RT, consequently causing a poor clinical outcome (Kim et al., 2019; Liu et al., 2020). Tumor cells that survive RT are transformed to a radioresistant phenotype, with an increased ability of invasion and metastasis (Kim et al., 2014; Liu et al., 2017). Therefore, clinical methods that increase ROS levels without causing hypoxia or alleviating hypoxia during RT, could greatly improve the therapeutic outcomes of RT.

Hypoxia is as an important characteristic of the tumor microenvironment caused by the consumption of oxygen by the rapidly growing tumor cells and insufficient oxygen supply (Peter and Arnulf, 2007; Masuda and Belmonte, 2013). Treatments that produce ROS and rely on ROS to be effective, such as RT or photodynamic therapy (PDT), can also cause hypoxia. Hypoxia markedly reduces the therapeutic effect of the treatments mentioned above (Peter and Arnulf, 2007; Yao et al., 2017); therefore, a series of measures have been explored to reverse or reduce the hypoxic environment (Huo et al., 2019). The main strategies involve the direct supply of oxygen (Xie et al., 2019), *in situ* production of oxygen (Chen et al., 2016), and the inhibition of oxygen consumption (Xia et al., 2019). Although these methods reduce the limitation of hypoxia on the therapeutic effect to some extent, they increase the complexity of the system, and the uncertainty of the treatment. A smarter system that not only reduces hypoxia during the treatment process but also enhances the treatment effect through adjuvant therapy, is of utmost importance. Thus, an effective tumor treatment should be explored in this direction.

Avoiding oxygen consumption by carrying oxygen (Cheng et al., 2015; Chen et al., 2017) or carrying ROS releasing groups (Mahmoud Asadirad et al., 2013; Kolemen et al., 2016) in the tumor microenvironment is an effective approach to overcome the insufficient oxygen supply at the tumor site and hypoxia caused during PDT. The release of ROS could not only overcome the limitation of hypoxia on PDT, but also solve the problem of insufficient *in vivo* penetration of PDT, since it is excited by visible light (Muhammad Idris et al., 2012).

**SCHEME 1:**
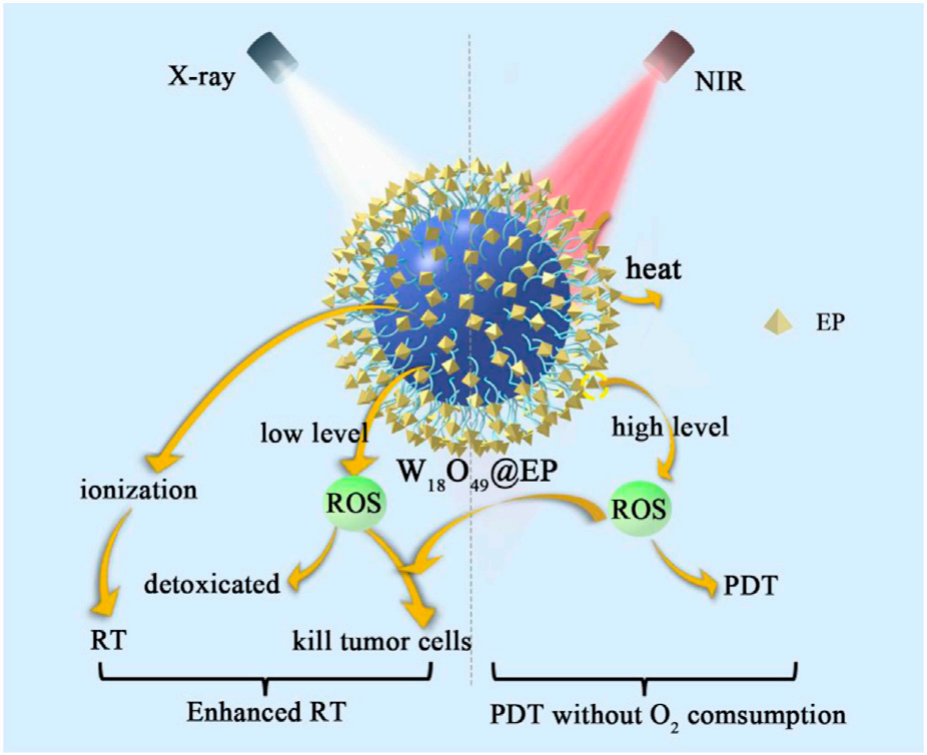
The mechanism of W_18_O_49_@EP under NIR and X-ray.

This work reports a combination of photothermal effect and RT that effectively reduced the hypoxic microenvironment of the tumor. W_18_O_49_ with dual effects of photothermal effect and RT sensitization was synthesized, and then the anthracene endoperoxide derivative compound (abbreviated as EP) that releases singlet oxygen in a thermal-controlled manner was covalently connected to it (Mahmoud Asadirad et al., 2013). As shown in Scheme 1, this system (named as W_18_O_49_@EP) has multiple advantages. First, it combined PDT with RT. Secondly, the ROS released by the photothermal effect was converted into oxygen *in vivo* to reduce the hypoxic microenvironment of the tumor. Thirdly, the singlet oxygen released by the photothermal effect synergistically acted with the ROS produced by RT to enhance the effect of ROS on inducing apoptosis. Finally, ROS were induced by NIR, which has a better penetration depth than visible light, the therapeutic effect on the tumor was enhanced through these multiple effects and an effective therapeutic protocol could be considered for an effective tumor therapy.

## 2 Experimental section

### 2.1 Materials

Tungsten hexachloride (WCl_6_), poly acrylic acid (PAA, Mw = 2000 D), diethylene glycol (DEG), ethyl dimethylaminopropyl carbodiimide (EDC) and sulfo-NHS were obtained from Sigma Aldrich (St. Louis, MO, United States). Methyl 4-(4,4,5,5-tetramethyl-1,3,2-dioxaborolan-2-yl)benzoate, methylene blue, 9-bromo-10-phenylanthracene, and H_2_N-PEG-OH (MW: 3,000 g/mol) were purchased from Sigma-Aldrich. All chemicals were analytically pure and used as purchased without further purification, unless otherwise noted.

### 2.2 Synthesis of W_18_WO_49_ nanoparticles

W_18_O_49_ NPs were prepared according to a procedure previously reported (Huo et al., 2014). Briefly, 500 mg WCl_6_ and 200 mg PAA were dissolved in 100 ml DEG. The mixture was stirred under the protection of N_2_ to fully dissolve the substances, and then placed at 180°C for approximately 30 min to allow the reaction. When the solution turned colorless to dark green, it was allowed to cool to room temperature. Subsequently, the mixture was centrifuged, the supernatant was removed and the pellet was dissolved in 50 ml deionized water. This procedure was repeated three times.

### 2.3 Synthesis of EP

Compound 3 was prepared according to a method previously reported (Mahmoud Asadirad et al., 2013; Kolemen et al., 2016). Next, compound 3 (50 mg, 0.12 mmol) and H_2_N-PEG-OH (292 mg, 0.086 mmol, MW: 3,000 g/mol) were dissolved in 5 ml dry tetrahydrofuran. N,N′-dicyclohexylcarbodiimide (26 mg, 0.12 mmol) and 4-dimethylaminopyridine (11 mg, 0.15 mmol) were added to the solution. The reaction mixture was stirred for 2 h until the formation of a precipitate. The precipitate was filtered and removed, and cold diethyl ether was added to the solution to precipitate the product (EP). Pure EP was filtered (187.1 mg, 42%).

### 2.4 Synthesis of W_18_O_49_@EP NPs

W_18_O_49_ (50 mg) NPs dissolved in PBS (10 ml) were activated with EDC (100 mg) and sulfo-NHS (50 mg). Subsequently, EP (75 mg) was added into the above solution. The mixture was allowed to react for 10 h at 4°C under vigorous stirring. Then, the as-obtained W_18_O_49_@EP NPs were harvested by three cycles of centrifugation and washing with PBS to remove the unreacted materials and excess of the reagents. Purified W_18_O_49_@EP NPs were re-suspended in PBS and stored at 4°C for further application.

### 2.5 Characterization

Images from transmission electron microscopy (TEM) were obtained using JEOL JEM-2010 (HR). Absorption spectra were recorded using a UV-Vis spectrophotometer (Persee DU1900, Beijing, China). The hydrodynamic particle size was characterized using dynamic light scattering (DLS) by ZetaSizer Nano-ZS90 (Malvern Instrument). The X-ray diffraction (XRD) pattern was recorded in the 2θ range of 20–60°.

### 2.6 Intracellular ROS

Human gastric carcinoma cells (MNK cells) were routinely cultured in Dulbecco’s Modified Eagle Medium (DMEM) at 37 and 5% CO_2_. Subsequently, they were cultured in a six-well plate to evaluate the ability of W_18_O_49_@EP NPs to produce intracellular ROS. When cells reached a confluence over 80%, the medium was removed and fresh medium containing the following substances was added: 1) PBS, without any treatment, used as control; 2) H_2_O_2_ (0.1 mM) without any other treatment; 3) PBS and laser irradiation (808 nm, 0.48 W/cm^2^) for 5 min; 4) W_18_O_49_ NPs (0.1 mg/ml) and laser irradiation (808 nm, 0.48 W/cm^2^) for 5 min; 5) W_18_O_49_@EP NPs (0.1 mg/ml) and laser irradiation (808 nm, 0.48 W/cm^2^) for 5 min. The fluorescent probe H_2_DCFDA was added after 12 h and incubated for 2 h. Confocal laser microscopy was used to evaluate the fluorescence of the cells after the treatments and images were collected.

### 2.7 *In vitro* cell experiment

The method used for this experiment was similar to the one above but using different groups. Cells were divided into six groups and treated as follows: 1) control: without any treatment; 2) W_18_O_49_ + RT: W_18_O_49_ NPs (0.1 mg/ml) and X-ray exposure (5 Gy) for 5 min; 3) W_18_O_49_ + Laser: W_18_O_49_ NPs (0.1 mg/ml) and laser irradiation (808 nm, 0.48 W/cm^2^) for 5 min; 4) W_18_O_49_@EP + Laser: W_18_O_49_@EP NPs (0.1 mg/ml) and laser irradiation (808 nm, 0.48 W/cm^2^) for 5 min; 5) W_18_O_49_ + Laser + RT: W_18_O_49_ NPs (0.1 mg/ml) and laser irradiation (808 nm, 0.48 W/cm^2^) simultaneous to X-ray exposure (5 Gy) for 5 min; 6) W_18_O_49_@EP + Laser + RT: W_18_O_49_@EP NPs (0.1 mg/ml) and laser irradiation (808 nm, 0.48 W/cm^2^) simultaneous to X-ray exposure (5 Gy) for 5 min. Different fluorescent probes were added after 12 h and incubated for 2 h. Confocal laser microscopy was used to evaluate the fluorescence of the cells after the treatments and images were collected.

### 2.8 Tumor model

A total of female BALB/c athymic nude mice, 6–8 week old, weighing 20 g were provided by the Comparative Medical Center of Yangzhou University (Yangzhou, China) and housed in a conventional clean, specific pathogen-free (SPF) facility, under a 12-h light/dark cycle with food and water at libitum, where food, water, bedding, and cages were irradiated before use. Room temperature and humidity at ∼25°C and ∼50% were continuously monitored. All procedures using animals were approved by the animal Protection Committee of Nanjing University (Nanjing, China). Next, 2 ×10^6^ hepatocellular carcinoma (HCC-4) cells suspended in 50 μl PBS were subcutaneously injected into the back of each mouse to establish the tumor model.

### 2.9 *In vivo* CT imaging

Mice were treated with 100 μL PBS, W_18_O_49_ (0.1 mg/ml), and W_18_O_49_@EP (0.1 mg/ml). The analysis was carried out at 6 h after injection using Hiscan XM Micro CT with X-ray tube settings at a current of 133 μA and a voltage of 60 kV (Suzhou Hiscan Information Technology, Jiangsu, China).

### 2.10 *In vivo* anti-tumor effect

When the tumor size reached approximately 100 mm^3^, mice were randomly divided into six groups (*n* = 5 per group). The treatment of the mice in each group was identical as in the *in vitro* experiment described in the paragraph 2.7. The body weight of the mice was recorded, and the tumor size was measured every 2 days. The tumor volume was calculated according to the following Eq. 1:

$$V=\frac{\left(L\times W^{2}\right)}{2}$$ (1)

where L is the shortest diameter of the tumor and W is the shortest diameter of the tumor.

### 2.11 Pathological analysis and hematological assay

Normal mice were intravenously (i.v.) injected with W_18_O_49_@EP NPs, sacrificed, and blood and major organs (kidney, lung, spleen, liver, and heart) were harvested at day 1, 7, and 21. They were fixed, embedded into paraffin, cut into sections, stained with H&E using a standard protocol, and visualized under light microscopy. Blood was collected in anticoagulant tubes containing sodium EDTA and separation gel were analyzed.

## 3 Results and discussion

### 3.1 Preparation and characterization of W_18_O_49_ NPs and W_18_O_49_@EP NPs

**SCHEME 2:**
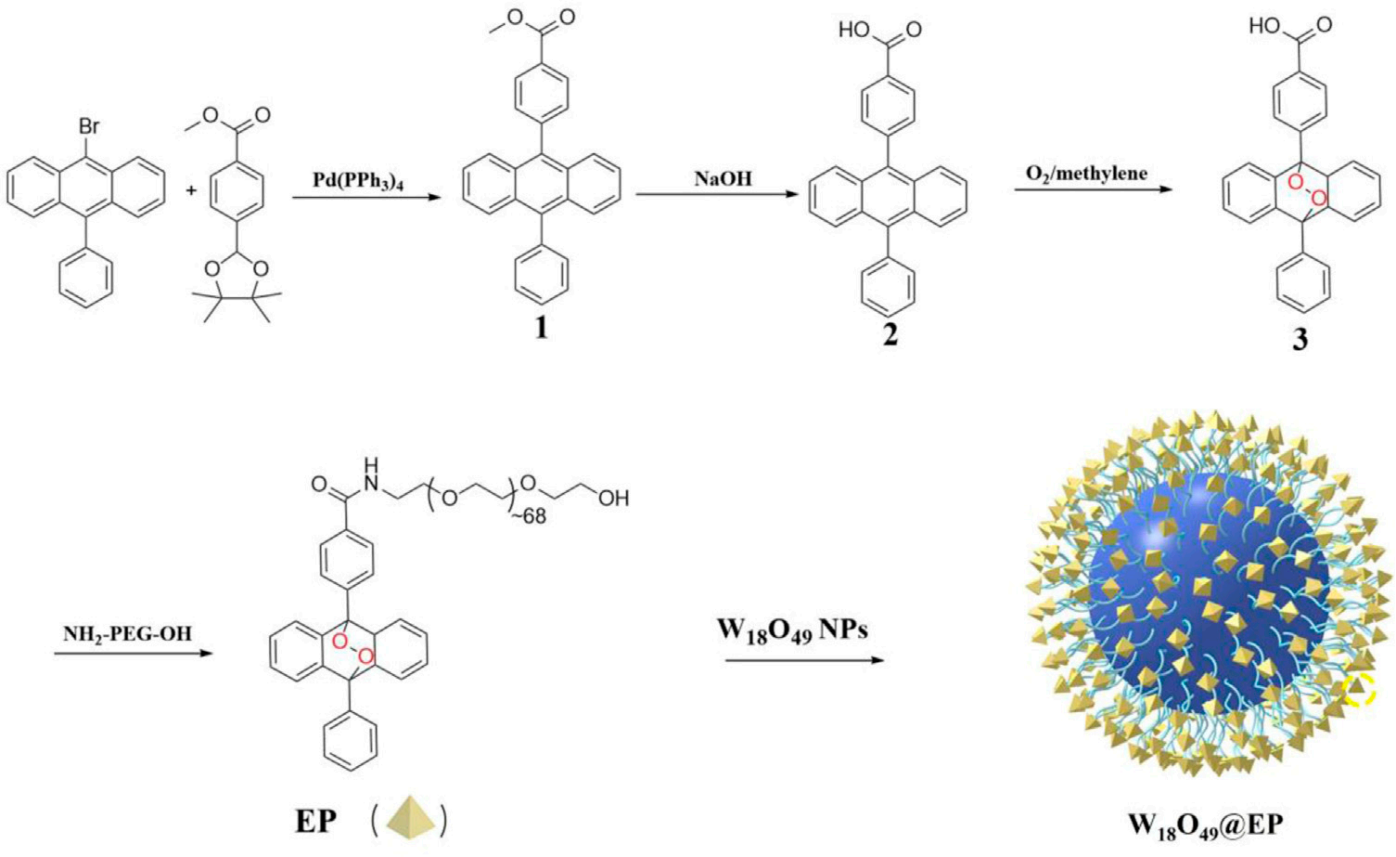
Synthetic route of W_18_O_49_@EP NPs.

W_18_O_49_ NPs were synthesized according to a method previously reported (Huo et al., 2014; Huo et al., 2017). W_18_O_49_@EP NPs were synthesized as shown in Scheme 2.

**FIGURE 1:**
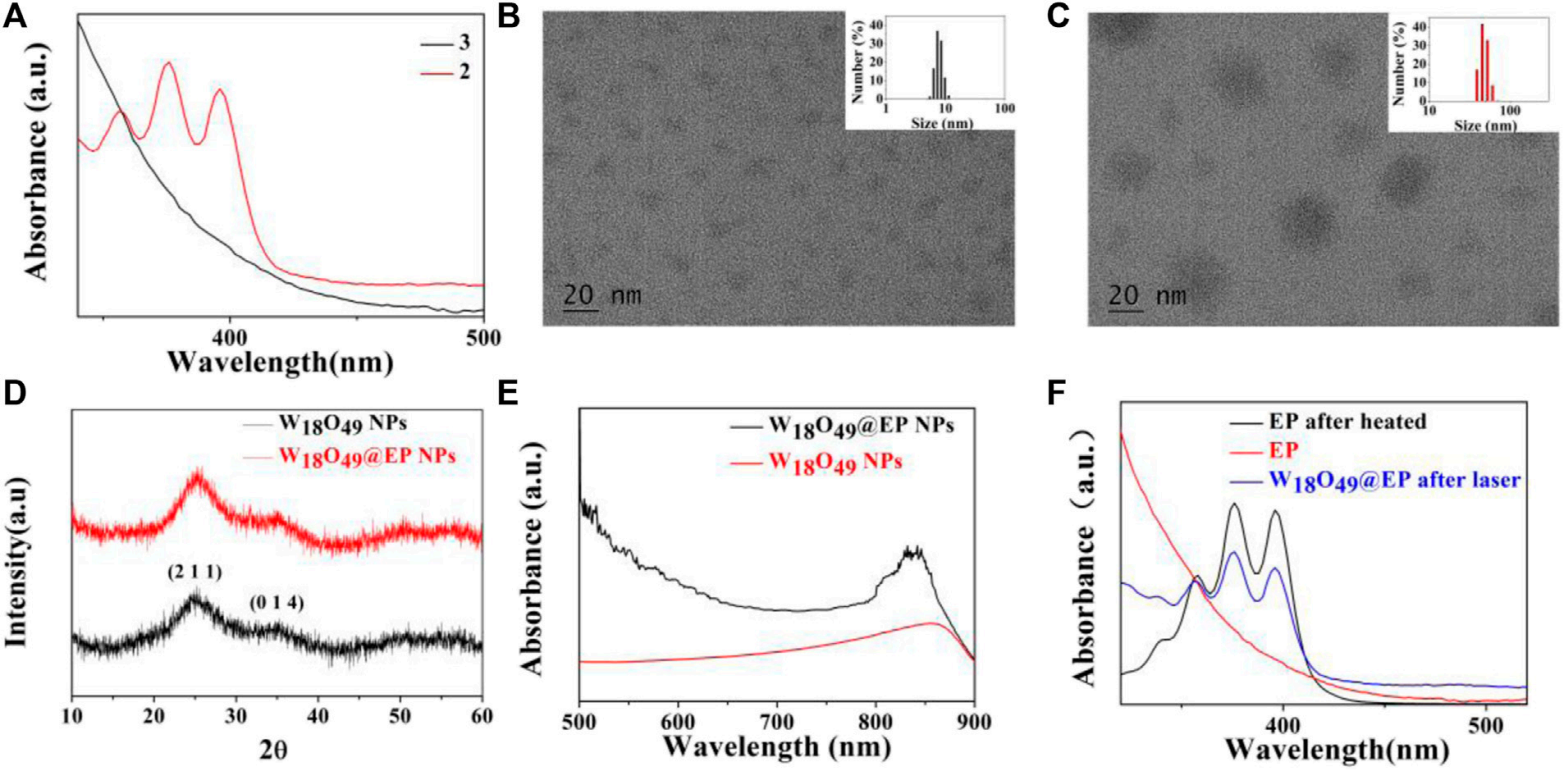
Characterization of materials. **(A)** UV-Vis spectra of compound 2 and 3. **(B)** TEM image of W_18_O_49_ NPs, (the upper right insert is its DLS result). **(C)** TEM image of W_18_O_49_@EP NPs, (the upper right insert is its DLS result). **(D)** XRD spectra of W_18_O_49_ NPs and W_18_O_49_@EP NPs. **(E)** UV-Vis-NIR spectra of W_18_O_49_ NPs and W_18_O_49_@EP NPs. **(F)** UV-Vis spectra of EP, as well as the product of EP after heating, and the product of W_18_O_49_@EP NPs after heating.

The introduction of a bridging oxygen structure (compound 2–3) was confirmed by UV-Vis spectra (Figure 1A). The disappearance of the absorption peaks at 358 nm, 376 nm, and 396 nm indicated the destruction of the anthracene ring as the introduction of the bridging oxygen (Chen et al., 2017). The morphology and size of W_18_O_49_ NPs and W_18_O_49_@EP NPs were analyzed by TEM (Figures 1B,C), DLS (Figures 1B,C insert), XRD (Figure 1D) and UV-Vis spectra (Figure 1E). W_18_O_49_ NPs had good mono-dispersibility in PBS with a size of approximately 10 nm according to TEM analysis, as shown in Figure 1B. DLS analysis indicated that W_18_O_49_ NPs were fairly uniform in size: their mean hydrodynamic diameter was 7.5 nm, with a poly-dispersity index of 0.132. W_18_O_49_ NPs were confirmed by XRD and UV-Vis-NIR spectrum analysis (Figures 1D,E) through the comparison with the data presented in a previous report (Kolemen et al., 2016). The diameter of W_18_O_49_@EP NPs increased after the modification of W_18_O_49_ NPs with EP and was approximately 25 nm as determined by the TEM images. The mean hydrodynamic diameter was 43.8 nm, with a poly-dispersity index of 0.108, which was bigger than that observed by TEM due to the influence of the surface organics. The XRD and UV-Vis-NIR spectrum of W_18_O_49_@EP NPs did not change (Figures 1D,E) compared with that of W_18_O_49_ NPs. EP had thermal response ability, thereby releasing its bridging oxygen, as shown in Figure 1F. A water bath at 65°C for 10 min resulted in the reappearance of the three absorption peaks that disappeared in Figure 1A, indicating that the bridging oxygen was released under the heating conditions and compound 3 was converted to compound 2. The three absorption peaks also reappeared in W_18_O_49_@EP NPs after 10 min of irradiation with an 808 nm laser, due to the photothermal conversion ability of W_18_O_49_ NPs (Kolemen et al., 2016).

**FIGURE 2:**
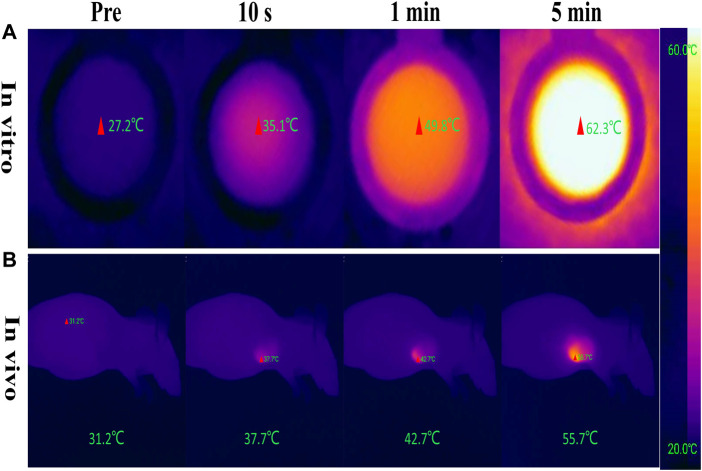
Photothermal conversion effect of W_18_O_49_@EP NPs. **(A)** Photothermal conversion effect of W_18_O_49_@EP solution (1 mg/ml) tested in a centrifuge tube; the data came from a thermal imaging camera. **(B)** A total of 0.1 ml W_18_O_49_@EP solution (10 mg/ml) was injected into the tumor of a tumor-bearing mouse; the data came from a thermal imaging camera. The laser irradiation was 808 nm and 0.48 W/cm^2^.

The photothermal conversion effect of W_18_O_49_@EP NPs using an 808 nm laser was evaluated to ensure the photothermal effect of W_18_O_49_@EP NPs. The *in vitro* results showed that the temperature of the W_18_O_49_@EP solution (1 mg/ml) rapidly increased under laser irradiation (808 nm, 0.48 W/cm^2^) from 27.2°C to 35.1°C in 10°s, reaching 62.3°C after 5 min (Figure 2A). Next, 0.1 ml W_18_O_49_@EP solution (10 mg/ml) was injected into the tumor of tumor-bearing mice. The temperature of the tumor changed after 30 min after laser irradiation (808 nm, 0.48 W/cm^2^) as monitored by a thermal imaging camera. The temperature at the tumor site increased from 31.2°C to 55.7°C within 5°min, indicating that W_18_O_49_@EP NPs possessed a photothermal therapeutic effect *in vivo* (Figure 2B). These results suggested that W_18_O_49_@EP NPs released enough heat under laser irradiation both *in vitro* and *in vivo* through the photothermal conversion effect, which was sufficient to release ROS by EP (Chen et al., 2017).

The above-mentioned experimental analysis showed the successful synthesis of W_18_O_49_@EP NPs, and the presence of EP did not affect the basic performance and photothermal conversion capacity of W_18_O_49_ NPs. The photothermal effect generated by W_18_O_49_ NPs was sufficient to release the loaded bridging oxygen by EP.

### 3.2 Ability of W_18_O_49_@EP NPs to release ROS

**FIGURE 3:**
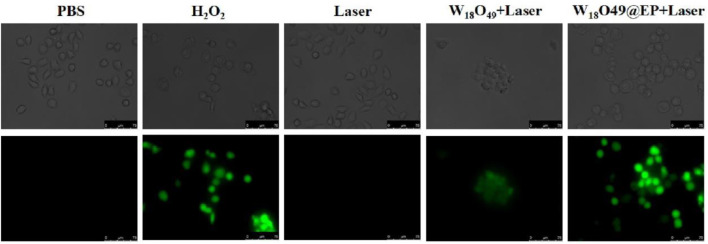
Intracellular production of ROS after different treatments.

Since the effectiveness of PDT relies on the action of ROS (Dougherty et al., 1998; Dolmans et al., 2003), the ROS producing ability plays a decisive role in the photodynamic effect. Therefore, the ROS production ability of W_18_O_49_@EP NPs was evaluated. The intracellular ROS producing ability of W_18_O_49_@EP NPs by H_2_DCFDA revealed that the combination of EP and W_18_O_49_ NPs greatly increased the amount of ROS (green fluorescence) produced under laser irradiation, which enhanced the PDT effect (Figure 3). In addition, cells were not able to detoxify and eliminate ROS through their self-regulation because of the increase in ROS content, and the effect of RT on tumor killing through ROS was increased, thus greatly enhancing the RT effect.

### 3.3 Anti-tumor effect *in vitro*

**FIGURE 4:**
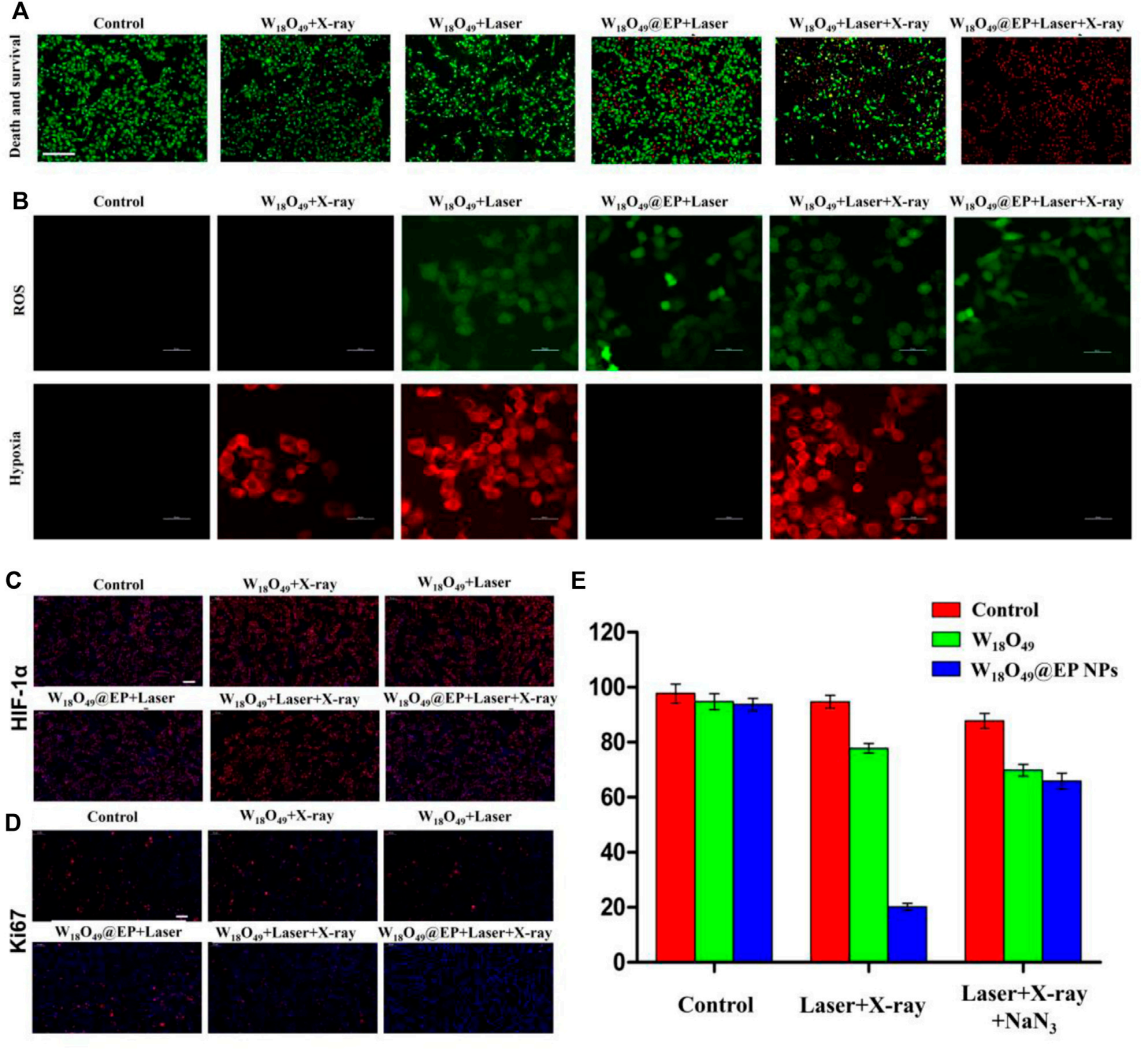
Anti-tumor experiment *in vitro*. **(A)** Cell death after different treatments. Red: dead cells; green: living cells.**(B)** Intracellular ROS content and hypoxia status after different treatments. **(C)** HIF-1 expression in cells after different treatments. HIF-1 is marked with red fluorescence. **(D)** Ki67 expression in cells after different treatments. Ki67 is marked with red fluorescence. **(E)** Cell survival after different treatments.

Only RT (W_18_O_49_+X-ray) and PDT (W_18_O_49_ + Laser) could kill a small number of cells without any additional oxygen supply, and the ability to kill cells was enhanced when a combination of RT and PDT (W_18_O_49_ + Laser + X-ray) was used (Figure 4A). However, almost all cells died after RT and PDT when the sensitizer W_18_O_49_ was replaced by W_18_O_49_@EP (W_18_O_49_@EP + Laser + X-ray), suggesting that W_18_O_49_@EP exerted a very significant effect when used in a combined treatment. This result might be due to the additional effects of EP in the material. The above-mentioned comparison experiments revealed that the ROS released by EP significantly enhanced the therapeutic effect of PDT and RT.

A series of *in vitro* experiments was performed to further elucidate the enhanced therapeutic effect of W_18_O_49_@EP NPs and to explore the underlying mechanism of action for its enhanced therapeutic effect. The amount of ROS production in the cells was significantly enhanced after the treatment with W_18_O_49_@EP NPs. Since ROS production was the main reason for RDT, the increased ROS production exerted an enhanced effect, representing an important aspect for the successful effect of RT (Figure 4A). Moreover, the hypoxia status in tumor cells was greatly improved after the treatment with W_18_O_49_@EP NPs, and almost no hypoxia was observed in the “W_18_O_49_@EP + Laser” group and “W_18_O_49_@EP + Laser + X-ray” group (Figure 4B). The hypoxia status after different treatments was also evaluated by staining the important hypoxia marker hypoxia inducible factor-1α (HIF-1α) (Dachs and Tozer, 2000; Liu et al., 2017), which further indicated that the hypoxia status after the treatment with W_18_O_49_@EP NPs was greatly reduced (Figure 4C). Since hypoxia is an important limiting factor for the effect of PDT and RT, overcoming hypoxia was another factor to consider for improving the treatment effect. Taken together, the therapeutic effect of W_18_O_49_@EP NPs was greatly improved through the above two approaches.

Ki67 is an important marker of tumor progression (Scholzen and Gerdes, 2000) to prove that our treatment involves the ability to inhibit the proliferation of the tumor (Figure 4D), and the results further proved that the treatment with W18O49@EP + Laser + X-ray effectively inhibited tumor proliferation. The combined treatment group (W_18_O_49_@EP + Laser + X-ray) was the one that showed the most reduced Ki67 expression, which proved that the combination treatment group was effective in inhibiting tumor proliferation.

Next, NaN_3_ was used to remove ROS to further confirm that the improvement of the treatment effect in the combination treatment group was mainly caused by an increase in ROS production during the treatment due to the introduction of EP. Our results showed that the treatment effect was greatly inhibited by adding NaN_3_ (Figure 4E), which further proved the role of ROS production in the combination therapy of RT and PDT. Therefore, W_18_O_49_@EP NPs, which were capable of self-supplying ROS, had a significant advantage in the combination therapy of RT and PDT.

### 3.4 CT contrast effect *in vivo*

**FIGURE 5:**
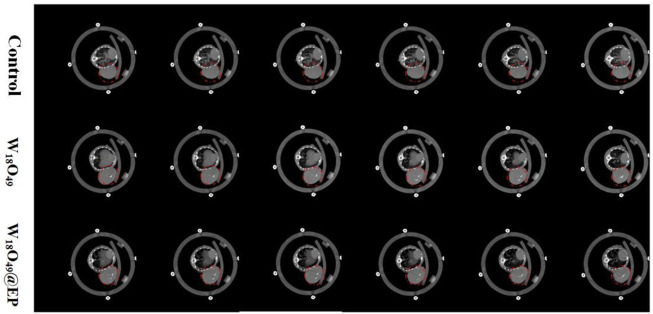
CT images of tumor-bearing mice after i.v. injection of 100 μL PBS, W_18_O_49_ (0.1 mg/ml) and W_18_O_49_@EP (0.1 mg/ml).

Since W_18_O_49_ NPs had a CT enhancement effect (Kolemen et al., 2016), both the W_18_O_49_ NPs and the W_18_O_49_@EP NPs were significantly enhanced at the tumor site, indicating that W_18_O_49_@EP NPs had enhanced CT angiography (Figure 5). These results also indicated that W_18_O_49_@EP NPs were enriched at the tumor site through passive targeting, thereby providing the possibility for subsequent *in vivo* treatment.

### 3.5 Anti-tumor effect *in vivo*

**FIGURE 6:**
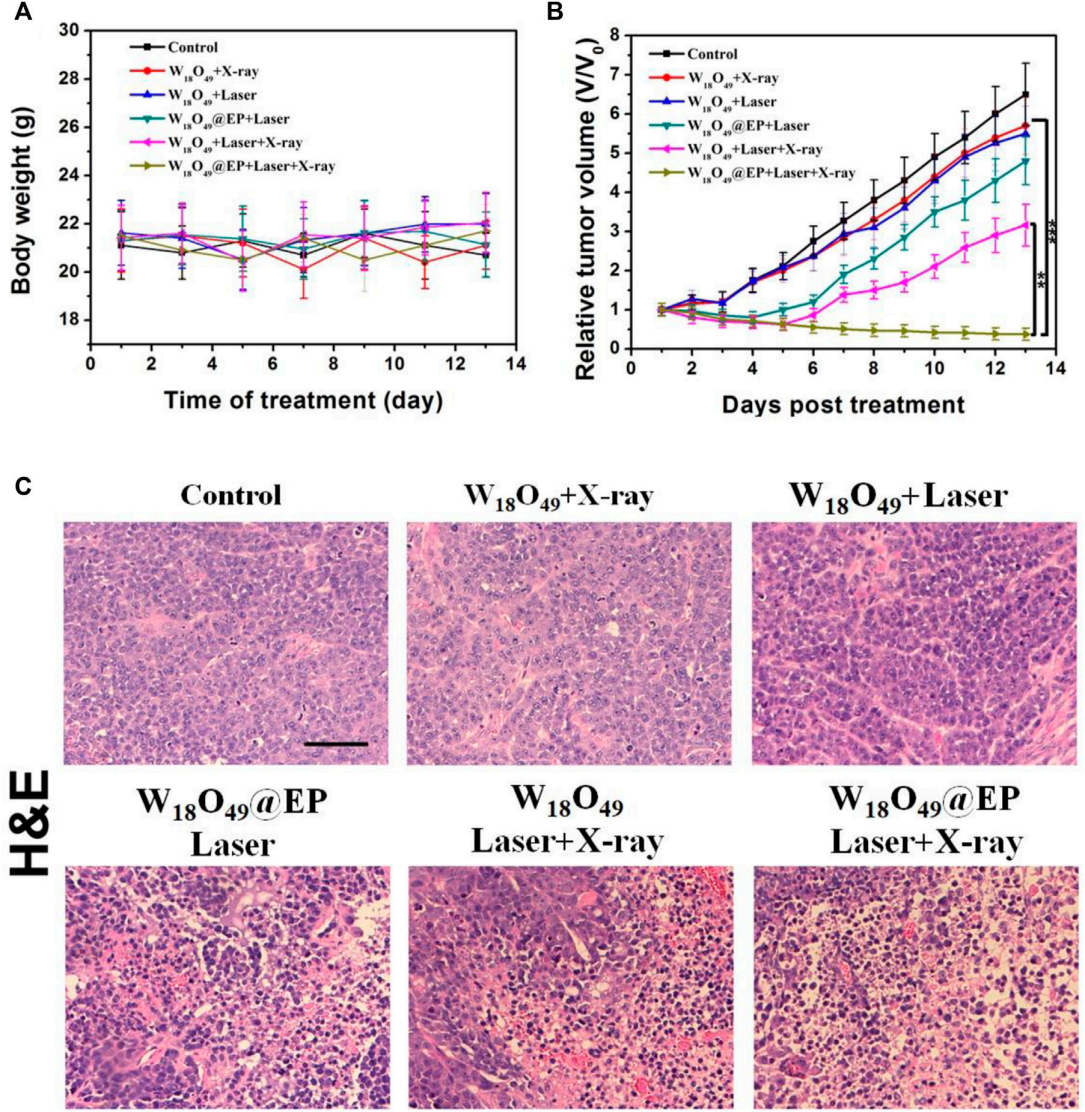
Anti-tumor effect *in vivo.* **(A)** Body weight of tumor-bearing mice after different treatments. **(B)** Tumor volumes of mice receiving different treatments. **(C)** Sections of tumor tissue after different treatments, stained by H&E.

The anti-tumor effect *in vivo* was evaluated on HCC-4 tumor bearing mice. After the tumor volume reached 100 mm^3^, mice were subjected to different treatments as shown in Figure 6. The body weight of all mice did not significantly change during the treatment (Figure 6A), which indicated that the treatment was not toxic to the mice. Figure 6B shows that the tumor volume after different treatments was reduced in the combination treatment group, and the tumor almost disappeared after 2 weeks of treatment. The tumor growth was inhibited to some extent in the other treatment groups, but the effect was not significant. Figure 6C shows the morphological changes of tumor tissues in different treatment groups. We found that in the ‘‘W_18_O_49_@EP Laser + X-ray” group, there was a lot of necrosis of the tumor cells, the cells were no longer tightly connected and the nuclei were huddled together. Indicating that the combination therapy was the most effective and effectively kills tumor cells. These findings were consistent with the results of the *in vitro* experiments, which also indicated that W_18_O_49_@EP NPs exerted an excellent therapeutic effect through the combination therapy.

### 3.6 Long-term pathological study

**FIGURE 7:**
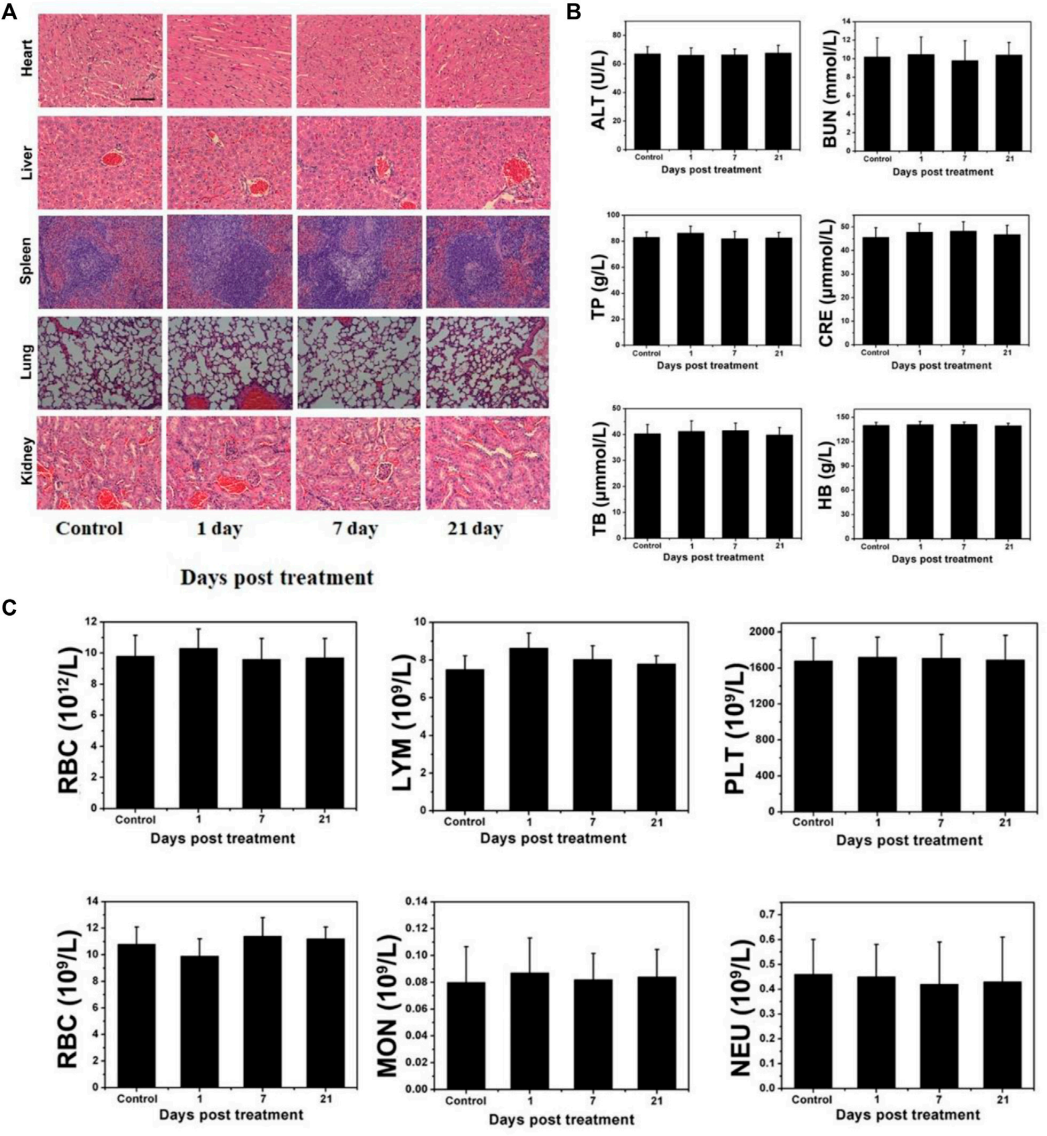
Long-term pathological study **(A)** Effect of W_18_O_49_@EP NPs in the main organs by H&E staining (kidney, lung, spleen, liver, and heart). **(B)** Serum biochemical study and hematological assay at day 1, 7, and 21. **(C)** Change in blood corpuscle at day 1, 7, and 21.

Although W_18_O_49_@EP NPs exerted a very good therapeutic effect both *in vitro* and *in vivo*, their long-term *in vivo* compatibility should be evaluated prior to clinical applications. Therefore, a pathological evaluation was performed on several major organs of normal mice that received W_18_O_49_@EP NPs to test their potential cytotoxicity. The morphology of cells in these five organs was hardly affected, indicating that the above NPs did not affect these organs (Figure 7A). In general, changes in blood cells are used to measure whether the various functions in the body are affected. The results showed that the treatment with these NPs hardly affected the content of various blood cells in the body of mice, as well as the function of some organs (Figures 7B,C). Thus, these results indicated that W18O49@EP was safe and feasible for *in vivo* treatment.

## 4 Conclusion

In conclusion, this work demonstrated that a novel NPs could provide ROS during RT and PDT for an effective anti-tumor therapy, effectively overcoming the problem of insufficient ROS production in PDT caused by an insufficient oxygen supply in tumors. Thus, the ROS production during the treatment enhanced the therapeutic effect. The effect of ROS in RT was highlighted with the increase in ROS production, which greatly enhances the RT effect of the tumor. This treatment strategy effectively overcame the hypoxia problem during the treatment, potentially preventing the poor prognosis caused by hypoxia. Therefore, the above-mentioned strategies could greatly improve the therapeutic effect on tumors, providing an effective approach for clinical anti-tumor studies.

## Data Availability

The raw data supporting the conclusions of this article will be made available by the authors, without undue reservation.
